# Supplementary material for: 1H-NMR-Based Metabolomics in Autism Spectrum Disorder and Pediatric Acute-Onset Neuropsychiatric Syndrome
Source: J Clin Med. 2022 Nov 1;11(21):6493. doi: 10.3390/jcm11216493 (PMC9658067; doi:10.3390/jcm11216493)
Supplement: Supplementary file 1 [file jcm-11-06493-s001.zip › jcm-1925989-supplementary.pdf]

Journal of
Clinical Medicine

Article

^{1}H-NMR-Based Metabolomics in Autism Spectrum Disorder and Pediatric Acute-Onset Neuropsychiatric Syndrome

Antonella Gagliano, Federica Murgia, Agata Maria Capodiferro, Marcello Giuseppe Tanca, Aran Hendren, Stella Giulia Falqui, Michela Aresti, Martina Comini, Sara Carucci, Eleonora Cocco, Lorena Lorefice, Michele Roccella, Luigi Vetri, Stefano Sotgiu, Alessandro Zuddas and Luigi Atzori

Supplementary Materials

1. Materials and Methods

1.2 Psychiatric evaluation

- **Pediatric anxiety Rating Scale (PARS)**, a clinician-rated instrument for assessing the severity of anxiety symptoms associated with anxiety disorders (social phobia, separation anxiety disorder, and generalized anxiety disorder) in children [79]

- **Pediatric Acute Neuropsychiatric Symptom Scale (PANSS)**, (Version: June 6, 2012), a parent version form to assess common PANDAS/PANS symptoms, developed based on the clinical experience of Swedo S., Kovacevic M., Latimer B. and Leckman J., with the help of Pohlman D., Moore K. and many other parents (2012) [2]. The structure of the scale provides for each symptom, included in the PANS criteria, a score from 0 to 5 points (none to very severe), with a total score range of 0–50 points. The impairment in self-esteem, family life, social acceptance, and school or job functioning, is estimated with a total score range of 0–50 points (no impairment to extreme difficulties).

- **Children's Yale-Brown Obsessive Compulsive Scale (CYBOCS),** a semistructured measure of obsessive-compulsive symptom severity in children and adolescents with obsessive-compulsive disorder (OCD) [80].

- **Yale Global Tic Severity Scale score (YGTSS),** a commonly used measure to document the intensity, complexity and the frequency of motor and phonic tics, performed by a clinician interview providing; the total score is a measure of overall tic severity [81].

- **Children's Global Assessment Scale (C-GAS),** a useful measure of overall severity of disturbance recommended to both clinicians and researchers as a complement to syndrome-specific scales [82].

- **UFMG (Universidade Federal de Minas Gerais) Sydenham'S Chorea Rating Scale (USCRS),** a scale designed to provide a detailed quantitative description of the performance of activities of daily living, behavioral abnormalities, and motor function of subjects with SC; the scale comprises 27 items and each one is scored from 0 (no symptom or sign) to 4 (severe disability or finding) [83]

- **Wechsler Intelligence Scale for Children, 4th Edn (WISC-IV),** the most widely used measure of intellectual ability for children from 6 to 16 years. It was developed to provide an overall measure of general cognitive ability (Full Scale Intelligence Quotient FSIQ), and also measures of intellectual functioning in Verbal Comprehension (VC), Perceptual Reasoning (PR), Working Memory (WM) and Processing Speed (PS) [84]

1.3 Sample preparation

Samples were thawed and treated with a modified Folch method to extract and separate hydrophilic and lipophilic metabolites. Four hundred mL of each serum sample were mixed with 600 mL of methanol, 600 mL of chloroform, and 175 mL of Milli-Q water. The samples were vortexed for 1 min and centrifuged for 30 min at 1700 g at room

temperature. Aliquots (10 mL) from each sample were used to create a pool for quality control (QC) samples. The QC samples were analyzed at the beginning and the end of the analysis. The hydrophilic and hydrophobic phases were obtained. The waterphase was divided into two aliquots and concentrated overnight using a speed vacuum centrifuge. For the 1H-NMR analysis, 700 mL of the water-phase containing low-weight molecules (amino acids, sugars, etc.) for each sample were concentrated overnight in a speed-vacuum. The concentrated water-phase was resuspended in 690 mL of D2O phosphate buffer (pH 7.4) and 10 mL trimethylsilyl propanoic acid (TSP) 5.07 mM. TSP was added to provide an internal reference for the chemical shifts (0 ppm). A total of 650 mL of the solution was transferred to a 5 mm NMR tube. The samples were analyzed with a Varian UNITY INOVA 500 spectrometer (Agilent Technologies, Inc., Santa Clara, CA, United States), which was operated at 499 MHz and equipped with a 5 mm triple resonance probe with z-axis pulsed field gradients and an auto-sampler with 50 locations. One dimensional 1H-NMR spectra were collected at 300 K with a pre-sat pulse sequence to suppress the residual water's signal. The spectra were recorded with a spectral width of 6,000 Hz; a frequency of 2 Hz; an acquisition time of 1.5 s; a relaxation delay of 2 ms; and a 90° pulse of 9.5 μs. The number of scans was 256. Each Free Induction Decay (FID) was zero-filled to 64 k points and multiplied by a 0.5 Hz exponential line broadening function [16].

After the instrumental analysis, the spectra were manually processed (phased and baseline corrected) by using MestReNova software (version 8.1, Mestrelab Research S.L.). More in detail, each NMR spectrum was divided into consecutive "bins" of 0.04 ppm. The resulting spectral area investigated was the region between 0.6 and 8.6 ppm. The regions between 4.60 and 5.2 ppm and between 5.24 and 6.6 ppm were excluded to remove variations of the residual water resonance and spectral regions of noise. The integrated area within each bin was normalized to a constant sum of 100. The final data set consisted of a 150 x 74 matrix. The columns represent the normalized area of each bin (variables), and the rows represent the samples (subjects).

1.4 Statistical Analysis

Multivariate statistical analysis was performed on NMR matrix by using SIMCA-P software (ver. 16.0, Sartorius Stedim Biotech, Umea, Sweden). Variables were Pareto scaled and then the initial data analyses were conducted using the Principal Component Analysis (PCA), to explore intrinsic clusters of the samples without classification and to find outliers. For this aim Hotelling's T2 test was applied. The PCA model was performed including the QC samples to evaluate the good quality of the analysis.

Then, supervised models were built. In particular, Partial Least Square Discriminant Analysis (PLS-DA) and Orthogonal Partial Least Square Discriminant Analysis (OPLS-DA) were applied. These two types of models maximize the discrimination between samples assigned to different classes and were employed to discriminate among patients with PANS, ASD and healthy subjects. The variance and the predictive ability (R2X, R2Y, Q2) were established to evaluate the strenght of the models. In addition, a permutation test (n = 400) was performed to validate each single model. This rigorous test compares the goodness of fit of the original model with that of randomly permuted models. Simultaneously, CV-ANOVA (analysis of variance testing of cross-validated predictive residuals) test was performed to determine significant differences between the different classes of the enrolled patients ($p < 0.05$). Variables corresponding to a VIP (Variables Important in the Projection) value of >1 (a measure of their relative influence on the model) from the supervised models together with the relative volcano plot were selected as the most important. To study a possible linear relationship between the metabolomic profile (matrix X, predictor variables, e.g. metabolites) and the clinical parameters (matrix Y, dependent variable) as measured by the psychodiagnostic instruments (PARS, PANSS, CYBOCS, YGTSS, C-GAS, WISC-IV-FSIQ and USCRS), Projection to Latent Structures (PLS) regression models were carried out. The selected variables were identified and quantified using the Chenomx NMR Suite 7.1 (Chenomx Inc., Edmonton, Alberta,

Canada). GraphPad Prism software (version 7.01, GraphPad Software, Inc., San Diego, CA, USA) was used to perform the univariate statistical analysis of the data.

To evaluate the significance of the metabolites resulting from the comparisons of the different classes of patients, U-Mann Whitney test was performed followed by the ROC curves analysis to test the sensitivity and specificity of the metabolites with p-values <0.05.

Metabolic pathways were generated using MetaboAnalyst 5.0, a web server designed to obtain information helpful in visualization and biological interpretation [www.metaboanalyst.ca].

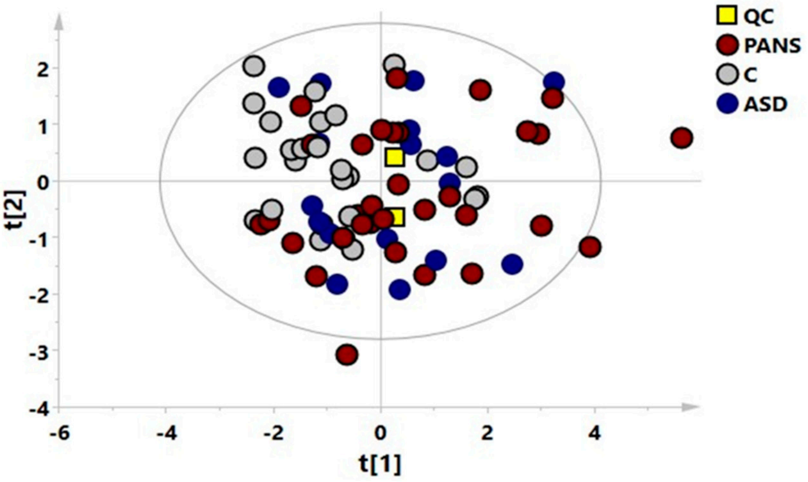

Figure S1. PCA scatter plot of the patients enrolled in the study.
Red circles = PANS patients; Blue circles = ASD patients; Grey circles = Controls; Yellow boxes = QC samples. Footnote: QC = Quality control; PANS = Pediatric Acute-onset Neuropsychiatric Syndrome; C = Controls; ASD = Autism Spectrum Disorder; QC = Quality control.

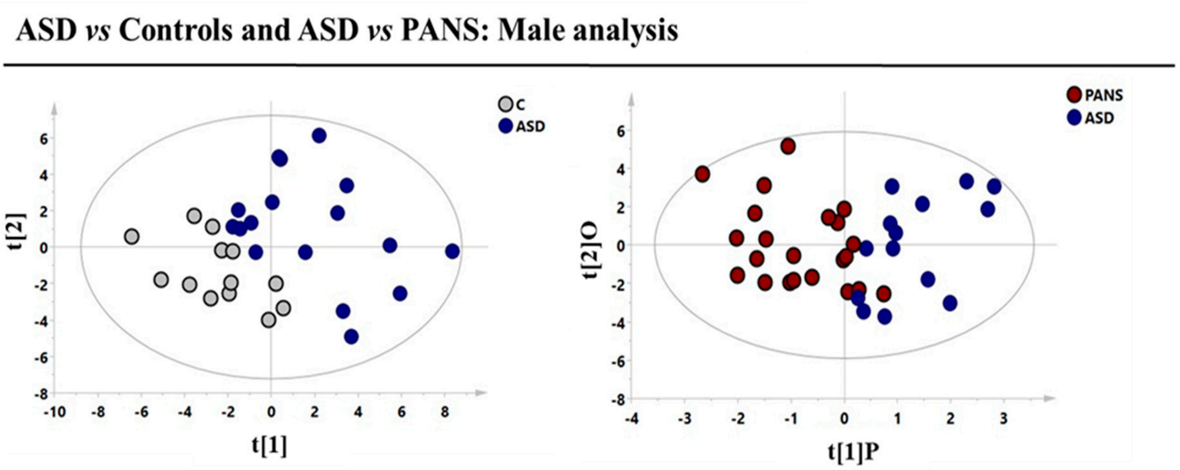

Figure S2. Multivariate analysis of the three classes of male subjects enrolled in the study: ASD vs Controls and ASD vs PANS.
Red circles = PANS patients; Blue circles = ASD patients; Grey circles = controls. Footnote: PANS = Pediatric Acute-onset Neuropsychiatric Syndrome; C = Controls; ASD = Autism Spectrum Disorder.
